# Current practice in the management of tetanus

**DOI:** 10.1186/cc13894

**Published:** 2014-05-27

**Authors:** Geeta M Govindaraj, Arakkal Riyaz

**Affiliations:** 1 Department of Pediatrics, Government Medical College, Kozhikode, Kerala, India

## Abstract

Tetanus is still a scourge among the under-privileged populations of the world, and unfortunately remains an important cause of death although a cheap, safe and highly efficacious vaccine is available. The rarity of the disease in some parts of the world results in newly trained physicians being unable to make a clinical diagnosis, and hampers the conduct of adequately powered randomized controlled trials. Several new and experimental pharmacological agents are being used to control the spasms in tetanus, and to combat the autonomic instability that occurs in the disease. New evidence is emerging regarding the use of antibiotics and intrathecal immunoglobulin in tetanus. It is imperative, therefore, that all physicians working in critical care should be aware of the current advances and evidence-based guidelines for management of tetanus in order to achieve the best outcomes, which Rodrigo and colleagues have reviewed in a recent issue of *Critical Care*.

## 

The past three decades have seen a marked reduction in the global burden of tetanus, and following the campaign directed at elimination of maternal and neonatal tetanus, the World Health Organization estimates that 58,000 newborns died in 2010, a 93% reduction from the situation in the late 1980s [1,2]. Several countries have successful immunization programs in place, but tetanus still continues to be an important, though hugely preventable, cause of mortality in several regions in the developing world [3,4]. Although tetanus toxoid was first used extensively in the Second World War, the types of wounds leading to tetanus have shown a recent trend to be less severe and often trivial, possibly because more serious wounds are generally given more expeditious and thorough medical attention [4].

The best chance of saving lives is by timely diagnosis and impeccable management of tetanus cases, which is a tough ask when physicians may not have experienced cases during their period of training. The issue is compounded by the fact that the diagnosis of tetanus is entirely clinical, with hardly any role for investigative modalities. Moreover, the management of tetanus is a challenge even for most physicians experienced in the care of such patients, and novices and old hands alike should be supported by having access to current recommendations and evidence-based therapeutic strategies.

The principles of management of tetanus include sedation and control of muscle spasms, neutralization of tetanus toxin, prevention of production of tetanus toxin by use of antibiotics to which *Clostridium tetani* is susceptible and by wound debridement, treatment of complications, including autonomic dysfunction, and supportive care [5].

The improvement of facilities in ICUs has led to a shift towards deep sedation, total paralysis and ventilator support in patients with severe tetanus. This has, however, resulted in a paradoxical increase in ventilator- associated complications, and renewed interest in sedation and control of muscle spasms by newer and often experimental pharmacological agents [6]. Moreover, facilities for ventilator support are often limited in the very same parts of the world where tetanus is still a bane to contend with.

The narrative review of Rodrigo and colleagues [1] takes a close look at the current evidence base for the use of newer agents for control of muscle spasms, including intravenous magnesium sulfate, intrathecal baclofen, dantrolene and botulinum toxin. The main stumbling blocks preventing widespread use of intrathecal baclofen were found to be the expensive intrathecal drug delivery system, the chance for infection and the need for specialized care in an ICU, due to its propensity to cause cardiorespiratory instability [7].

Autonomic dysfunction is an important problem to be tackled in patients with tetanus, since it may lead to a fatal outcome if not addressed adequately. Rodrigo and colleagues examine the current knowledge on the use of drugs for this purpose, including intravenous morphine, clonidine, labetalol and magnesium sulfate.

Several studies have looked into the route of administration of tetanus immunoglobulin [8,9] and Rodrigo and colleagues review the role of intrathecal immunoglobulin in minute detail, and presents a bird’s eye view of current thinking. The role of antibiotics is also focused on, and the advantages of using metronidazole over crystalline penicillin are clearly spelt out.

The evidence-based review by Rodrigo and colleagues was hampered by the varying scales used to classify the severity of tetanus in different studies, and the lack of firmly grounded evidence for use of time-tested therapeutic modalities, including benzodiazepines and antibiotics. The paucity of randomized controlled trials due to the rarity of the illness and the ethical concerns in withholding time-tested therapeutic modalities also came into sharp focus. Since the focus is now on the elimination of maternal and neonatal tetanus, the future will see a shift toward tetanus due to non-obstetric causes, including mass casualties. This includes iatrogenic causes, including surgery under non-sterile conditions, piercing of body parts, ritual scarification and injections by drug abusers. The excessive use of muscle relaxants by physicians not adequately adept in their use is likely to result in an unfortunate increase in morbidity and mortality as well.

## Conclusion

The most important factor that determines the outcome in tetanus is undoubtedly the quality of supportive care and the rapidity of initiation of treatment after a diagnosis has been made. Rodrigo and colleagues’ systematic narrative review on the pharmacological management of tetanus is a timely and exhaustive resource that will benefit clinicians, researchers and students, and will go a long way to ensuring that patients with tetanus get the benefit of optimal management and, hence, the best chance for intact survival.

## Competing interests

The authors declare that they have no competing interests.

## Authors’ contributions

GMG wrote the final draft of the paper. AR reviewed the final draft and suggested modifications. Both authors read and approved the final manuscript.
